# Mitogen-Activated Protein Kinase Phosphatases: No Longer Undruggable?

**DOI:** 10.1146/annurev-pharmtox-051921-121923

**Published:** 2023-01-20

**Authors:** Shanelle R. Shillingford, Anton M. Bennett

**Affiliations:** 1 Department of Pharmacology, Yale University School of Medicine, New Haven, Connecticut, USA; 2 Department of Chemistry, Yale University, New Haven, Connecticut, USA; 3 Yale Center for Molecular and Systems Metabolism, Yale University School of Medicine, New Haven, Connecticut, USA

**Keywords:** mitogen-activated protein kinase, protein tyrosine phosphatases, dual specificity protein phosphatases, allosteric site, small molecule inhibitors, cell signaling

## Abstract

Phosphatases and kinases maintain an equilibrium of dephosphorylated and phosphorylated proteins, respectively, that are required for critical cellular functions. Imbalance in this equilibrium or irregularity in their function causes unfavorable cellular effects that have been implicated in the development of numerous diseases. Protein tyrosine phosphatases (PTPs) catalyze the dephosphorylation of protein substrates on tyrosine residues, and their involvement in cell signaling and diseases such as cancer and inflammatory and metabolic diseases has made them attractive therapeutic targets. However, PTPs have proved challenging in therapeutics development, garnering them the unfavorable reputation of being undruggable. Nonetheless, great strides have been made toward the inhibition of PTPs over the past decade. Here, we discuss the advancement in small-molecule inhibition for the PTP subfamily known as the mitogen-activated protein kinase (MAPK) phosphatases (MKPs). We review strategies and inhibitor discovery tools that have proven successful for small-molecule inhibition of the MKPs and discuss what the future of MKP inhibition potentially might yield.

## THE DUAL-SPECIFICITY PHOSPHATASES: MKPs

Phosphorylation is one of the most common posttranslational modifications undergone by biomolecules and it controls the functional status of proteins, lipids, and carbohydrates. While kinases are responsible for the addition of phosphate, its removal is typically performed by a group of enzymes known as phosphatases (1). These phosphate groups are attached and removed on either a threonine, serine, or tyrosine residue (2) through catalysis by threonine/serine kinases and phosphatases, tyrosine kinases and phosphatases (3), or dual-specificity kinases and phosphatases (4, 5). The protein tyrosine phosphatase (PTP) superfamily is a group of approximately 100 enzymes in the human genome that comprise phosphotyrosyl-specific PTPs and dual-specificity phosphatases (DUSPs) (for reviews that provide a comprehensive breakdown of the classes and functions of these PTPs, see 6–8). Here, we focus on a specific group within the class of DUSPs (9–11) known as the mitogen-activated protein kinase (MAPK) phosphatases (MKPs) (12, 13).

**Figure 1:**
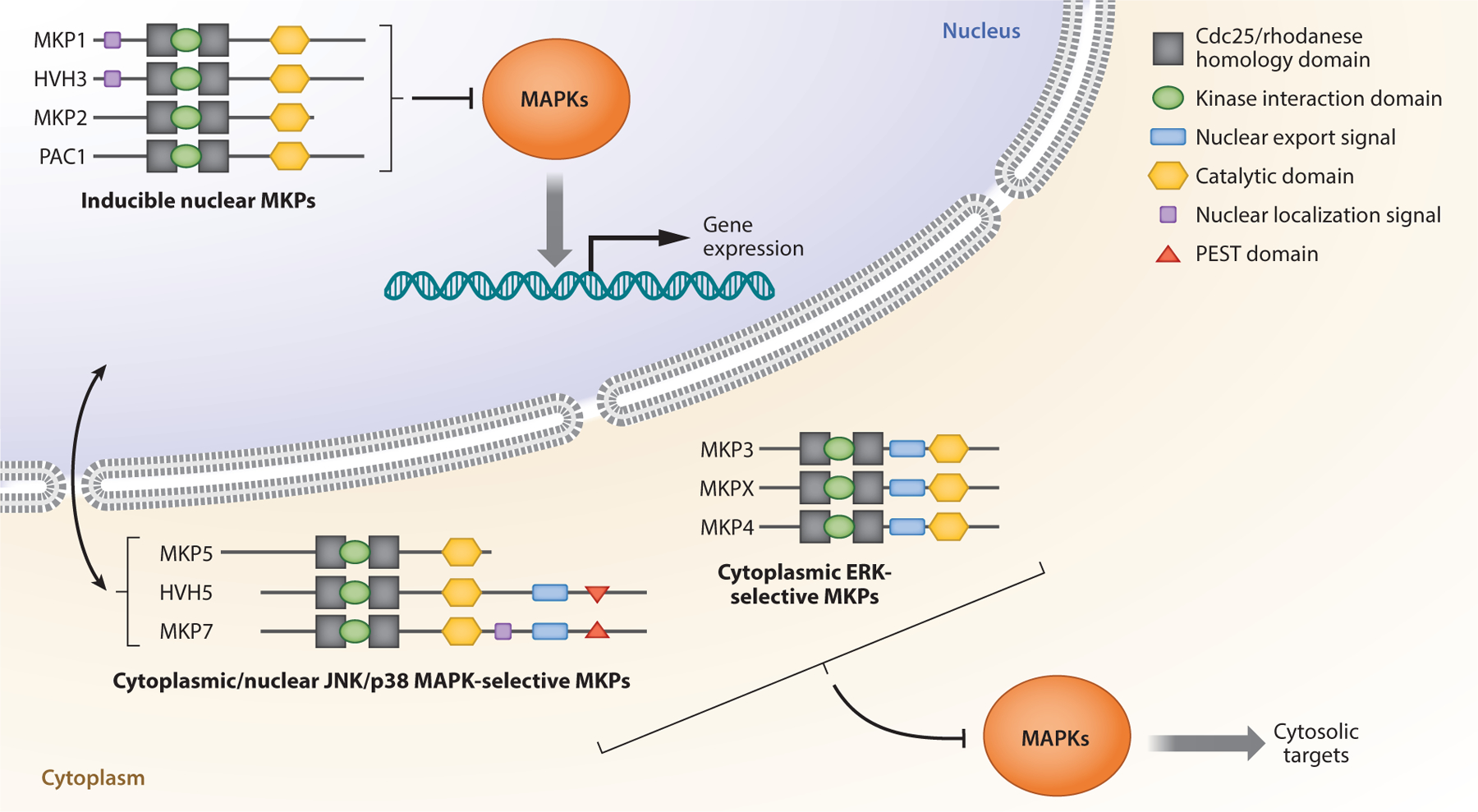
MKP pathway. The schematic shows the MKPs in the MAPK pathway. MKPs are either exclusively nuclear or cytoplasmic, and others such as MKP5, HVH5, and MKP7 shuttle between the cytoplasm and nucleus. MKPs contain noncatalytic domains that flank a conserved PTP domain. These noncatalytic domains direct MAPK interactions and subcellular localization. Abbreviations: ERK, extracellular signal–regulated kinase; JNK, c-Jun N-terminal kinase; MAPK, mitogen-activated protein kinase; MKP, MAPK phosphatase; PEST, peptide sequence rich in proline (P), glutamic acid (E), serine (S), and threonine (T); PTP, protein tyrosine phosphatase. Figure adapted with permission from Reference 13; copyright 2013 John Wiley & Sons.

The MKPs belong to a subgroup of DUSPs that are responsible for the dephosphorylation of extracellular signal–regulated kinases 1 and 2 (ERK1/2), p38 MAPK, and c-Jun N-terminal kinases (JNKs) at their phosphothreonine and phosphotyrosine residues within the signature TXY activation motif (12, 14). The MKPs are defined because of their exquisite substrate selectivity for the MAPKs and their high sequence similarity and domain homology (12) (Figure 1). Initially, the MKPs were considered to be MAPK regulatory enzymes with redundant functions (15); however, their MAPK substrates are essential for numerous critical cellular functions, including cell proliferation, apoptosis, cell growth, and cell differentiation (16). Thus, the MAPKs have been actively interrogated as therapeutic targets due to their aberrant signaling in diseases such as cancer, inflammatory and neurological diseases, and diabetes for decades (17–21) and as such have several successful inhibitors (17–21).

The MKPs are involved in the development of several diseases due, in most cases, to their increased expression and/or activation in certain tissues (22, 23). For instance, the first identified MKP, MKP1, which preferentially dephosphorylates p38 MAPK ≈ JNK ≫ ERK1/2 (24–26), is overexpressed in several cancers such as breast, lung, prostate, and ovarian cancers (27–29) (Table 1). In addition, MKP1 overexpression can confer resistance to lung cancer cells treated with cisplatin (29). These observations suggest that inhibition of MKP1 in combination with chemotherapeutic agents might enhance lung cancer cell death, thereby providing more effective therapeutic efficacy. MKP1 is also upregulated in the fat and skeletal muscle of obese nondiabetic subjects (30, 31) and in the livers of either obese mice fed a high-fat diet or in mouse models of genetic obesity (30, 31). MKP1 overexpression is suggested to be linked to metabolic dysfunction in the liver, where it is proposed to promote fatty liver disease (23, 32, 33). Thus, MKP1 inhibition is a possible target for the treatment of liver disease (34). The MKPs have been linked to innate and adaptive immunity defects and inflammatory and fibrotic disease (23, 32, 33) (Table 1). Recent work on MKP5 has demonstrated that it is involved in the activation of the transforming growth factor-beta (TGF-β) pathway, which, when promiscuously activated, causes tissue fibrosis. Mice lacking MKP5 are protected from fibrosis in multiple tissues, demonstrating that MKP5 is a target for the treatment of this devastating disease, which is associated with nearly 45% of deaths worldwide (35–37). While some MKPs, when overexpressed, are correlated with the progression of certain cancers and metabolic diseases, loss of MKP function can exacerbate inflammatory responses such as sepsis, as is the case for MKP2 (Table 1). Thus, in some cases, activating the MKPs, which is challenging, could offer an approach to dampen inflammatory responses. However, this type of approach has not been undertaken, thus the review focuses on the progress made in the development of MKP inhibitors. There is now overwhelming evidence highlighting the importance of MKP function in physiological and pathophysiological signaling, prompting widespread interest in the MKPs as therapeutic targets (38–40).

Certain challenges have slowed the progression in developing high-potency inhibitors for MKPs, and for PTPs in general (41, 42). Here, we discuss the mechanism of action of MKPs, the current challenges in the use of small-molecule MKP inhibition, and the progress that has been made toward developing MKP inhibitors. The current state of MKP inhibitor development suggests that much progress has been made and that the development of potent, highly specific MKP inhibitors is possible.

## MECHANISMS OF MKP CATALYSIS

**Figure 2:**
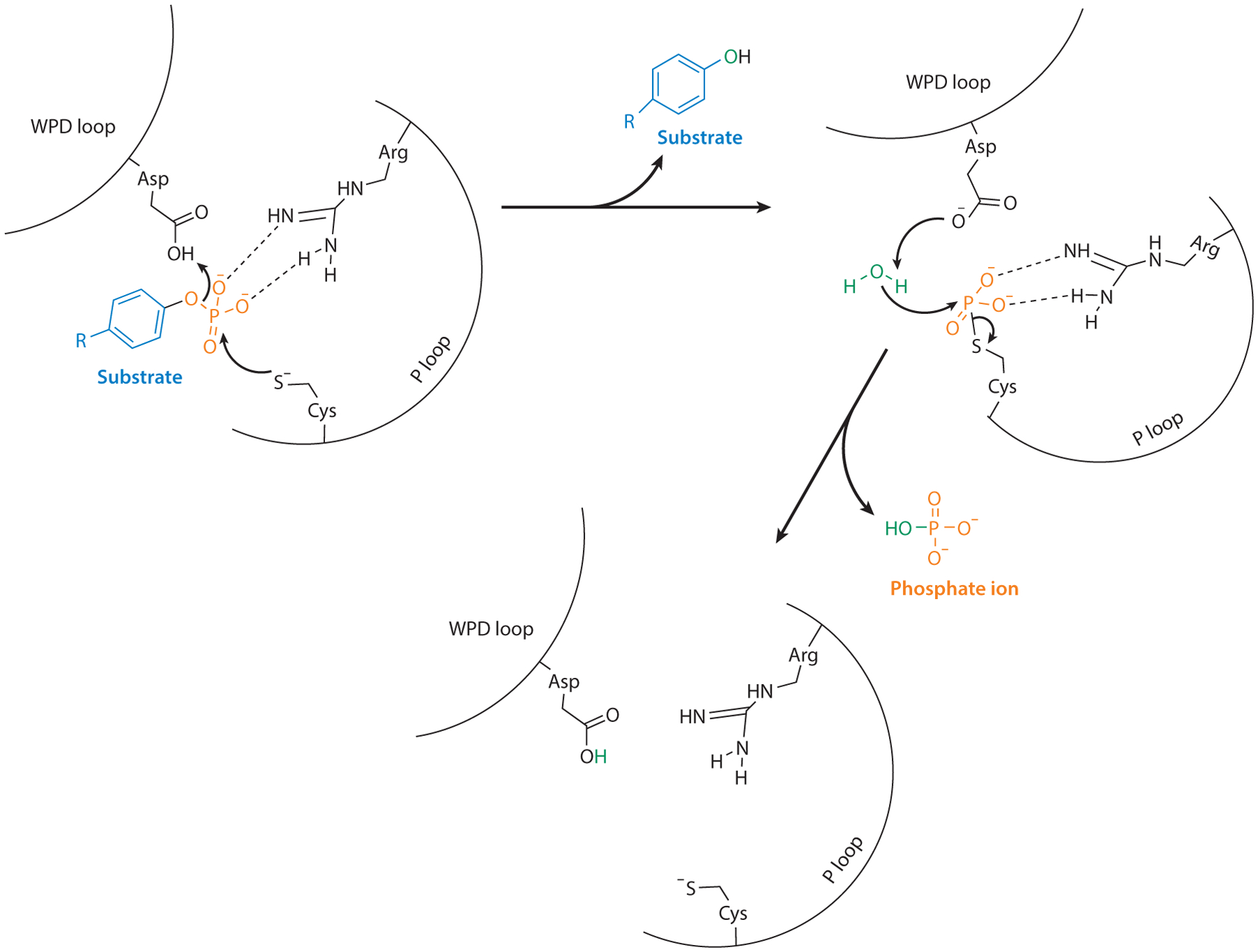
Catalytic mechanism of protein tyrosine phosphatases (PTPs). Scheme shows the proposed two-step mechanism by which PTPs utilize key residues in their active site to facilitate dephosphorylation of their substrates, and the proposed intermediate state before catalysis is complete. Figure adapted with permission from Reference 43; copyright 2013 Springer Nature.

The MKPs have an invariable catalytic sequence of **D**X_26_(V/L)X(V/I)H**C**XAG(I/V)-S**R**SXT(I/V)XXAY(L/I)M, where X is any amino acid and the boldfaced residues are critical for MKP catalysis (12). The H**C**-XXXXX-**R**S motif is shared by all PTPs (22, 43). The crystal structures for several of the MKPs have provided insight into the mechanism that MKPs utilize to catalyze MAPK dephosphorylation. The MKPs share a kinase-binding domain (KBD) for substrate interactions as well as the catalytic PTP domain, with a few MKPs having nonconserved N or C termini (23) (Figure 1). MKP crystal structures have revealed that key active site residues involved in catalysis are located within a phosphate-binding (P) loop and a general acid (WPD) loop in the PTP active site (43). The dephosphorylation of incoming substrates in the MKPs is a two-step process (Figure 2). The initial step is the nucleophilic attack by the catalytic cysteine thiolate group, which maintains a negative charge due to an acidic pK_a_ (44). This attack by the cysteine in the P loop is accompanied by an aspartic acid in the WPD loop, acting as a general acid, that donates a proton/hydrogen to the oxygen on the substrate-leaving group (12, 44). This two-pronged attack by cysteine and aspartic acid is paired with a deepening of the catalytic pocket through a conformational change that is required to accommodate the substrate in the MKPs (12). MKPs such as MKP1, MKP3, and MKP4 attain this structural change after substrate binding at the KBD, which results in the acquisition of an active conformation that promotes the nucleophilic attack by the cysteine (45–47). Following release of the dephosphorylated substrate, the phospho-thiolate intermediate is stabilized in the MKP, often by a neighboring arginine in the P loop via hydrogen bonding. The aspartate residue now acts as a general base, removing a proton from a nearby water molecule, which leads to this activated water molecule attacking the phosphate group and results in a phosphate ion being released, reverting the MKP to its prebound substrate state (Figure 2).

## THE MKP INHIBITOR CHALLENGE

Uncertainty in considering the MKPs as therapeutic targets emerged because of their high sequence homology, particularly within their active site. The MKPs typically share 37–50% sequence similarity in the catalytic domain among its members, although some have as much as 75% similarity between each other. This highly conserved sequence and structure among the MKPs makes MKP inhibitor development challenging. Additionally, the typical strategy of identifying a small-molecule inhibitor often involves a high-throughput screen (HTS) of existing chemical libraries (48). However, the presence of the charged active-site cysteine often leads to indiscriminate hits such as acid halides, quinones, and peroxides that interact with the cysteine thiolate (49, 50) or genuine hits with charged functional groups that give rise to poor bioavailability (42). Also, the cysteine thiolate is prone to oxidation via reactive oxygen species (39). Use of purified PTPs in in vitro assays, especially with reactive molecules, can oxidize the cysteine and inactivate the phosphatase, which leads to poor-quality hits being identified.

While the apo-crystal structures of the PTP domains from several MKPs have been solved (46, 47, 51–55), many remain unsolved, making it challenging to fully characterize their mechanism of action and to apply in silico structure-based strategies in the drug discovery and development process. Indeed, domain homology modeling is quite tractable in this regard, as in silico drug modeling will become more accurate with an increasing number of solved MKP structures (56). However, there are likely to be nuances in the structural characteristics of each MKP that are missed with homology modeling. In addition, cocrystal structures of the MKPs in complex with small-molecule inhibitors are rare. Thus, even when there are initial inhibitor hits, the lack of a crystal structure makes it challenging to delve into defining the critical residues involved in inhibitor action. Despite these challenges, MKP small-molecule inhibition is under active investigation. We discuss below some of the major breakthroughs in MKP inhibition and strategies that have proven most effective in finding small-molecule inhibitors.

### MKP1 (*DUSP1*)

MKP1, which dephosphorylates p38 MAPK and JNK, with lesser potency to ERK, is overexpressed in cancer and in metabolic syndrome (28) (Table 1). While the identification of highly potent, specific MKP1 inhibitors has not been fully realized, it is still viewed as a very attractive therapeutic target for the treatment of these diseases.

### Sanguinarine and Chelerythrine

**Figure 3:**
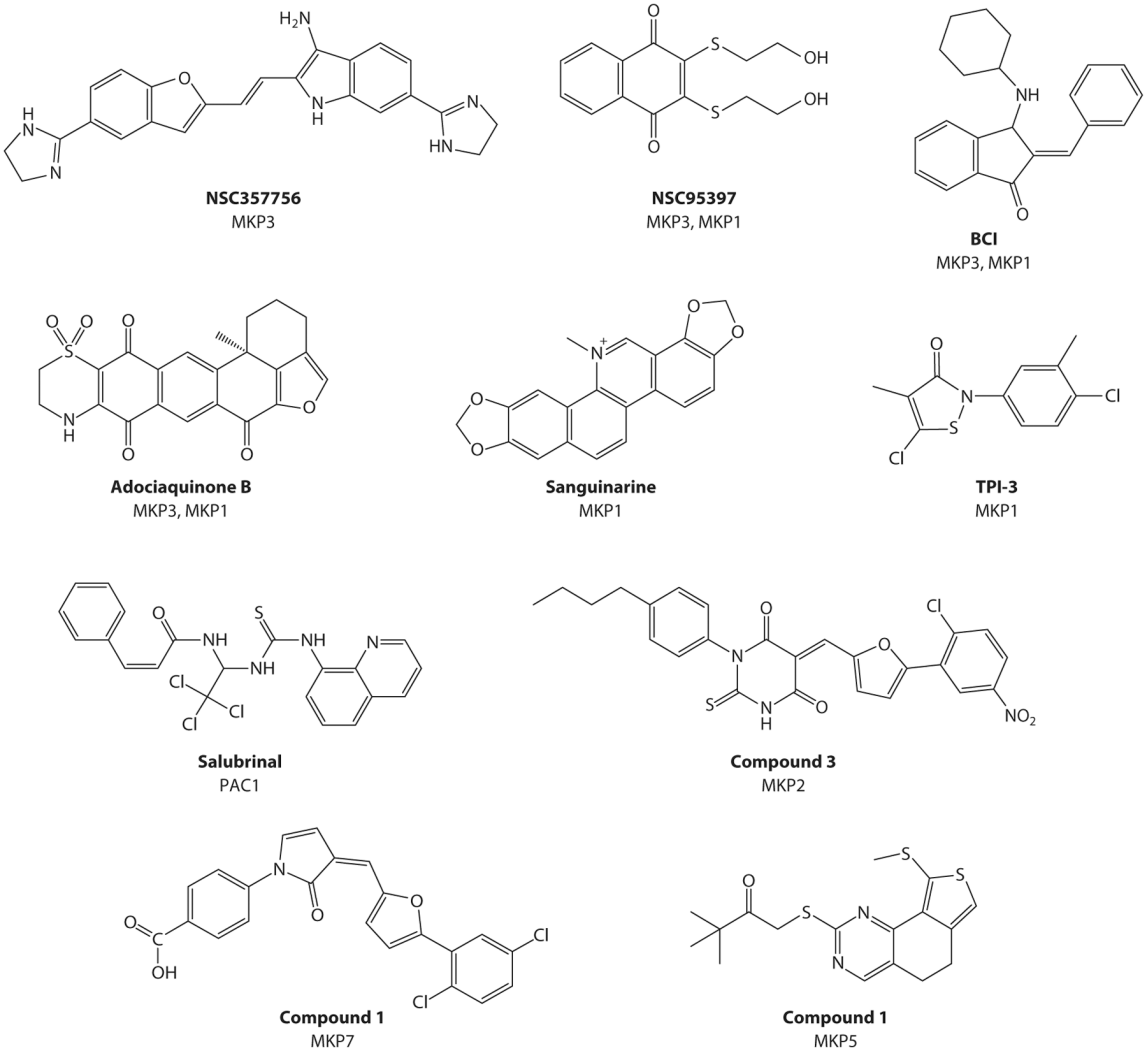
Chemical structures of mitogen-activated protein kinase phosphatase (MKP) inhibitors. Chemical structures are given for the main pharmacophores that have emerged in a variety of screens for MKP inhibitors, including NSC357756 (75), NSC95397 (78), BCI (80), adociaquinone B (84), sanguinarine (86), TPI-3 (88), salubrinal (93), Compound 3 (98), and Compound 1 (105, 109).

In a HTS based on the regulation of ERK activity in intact cells, Vogt et al. (57) identified the natural plant product sanguinarine and its derivative chelerythrine as MKP1 inhibitors. This assay was developed for MKP1 by transiently transfecting cells with full-length MKP1 and then stimulating the ERK pathway with 12-*O*-tetradecanoylphorbol-13-acetate (TPA), with and without compounds, followed by a fluorescent readout of ERK activity. This screen utilized a library of 720 commercially available natural products. Of these, 22 compounds showed increased ERK activity in MKP1-expressing cells, and of the 22, only sanguinarine (ED_50_ = 10 μM) showed increased levels of activated ERK. Use of MKP3 in a similar chemical screen showed that in MKP3-expressing cells, sanguinarine had a negligible effect, indicating specificity for MKP1 over MKP3. To confirm sanguinarine’s specificity for MKP1 over other PTPs, in vitro assays were performed with MKP3, Cdc25B, VHR, PTP1B, and MKP-L. MKP1 had an IC_50_ (17.3 ± 1.2 μM) that was more than three- to fivefold lower than that of MKP3, Cdc25B, VHR, and PTP1B, while a close relative, MKP-L, had a slightly lower IC_50_ of 12.5 ± 2.1 μM (57). Testing both MKP1 and MKP-L against other commercially available sanguinarine derivatives revealed one other potent inhibitor, chelerythrine, which preferentially inhibited MKP1 (IC_50_ = 16.2 ± 1.7 μM) over MKP3 (IC_50_ = 26.3 ± 7.6 μM). Both sanguinarine and chelerythrine are known to have cytotoxic effects in both healthy and cancer cells (58). However, the concentrations at which they are used to inhibit MKP1 are much lower than that which induces these effects (57). The authors also demonstrated that in sanguinarine and its analogs, the benzophenanthridine pharmacophore is important for MKP1 inhibition (Figure 3). While the mechanism through which sanguinarine and its analogs inhibit MKP1 is unknown, the benzophenanthridine pharmacophore can be used as a basis for structure-activity relationship (SAR) studies for the development of more potent and selective MKP1 inhibitors (57). Despite these data, it is highly unlikely that either sanguinarine or chelerythrine exerts MKP-specific effects to be useful tool compounds, given that these compounds exhibit numerous cellular responses on many other targets (59, 60).

### Tyrosine Phosphatase Inhibitor-2 and 3

An in vitro assay using a chemical compound library from ChemBridge identified a small biomolecule, which the authors referred to as tyrosine phosphatase inhibitor (TPI)-2, as a lead hit that decreased MKP1 activity (61). Subsequently, six of its analogs were commercially purchased to determine the effects on MKP1, which led to the discovery of an analog with similar in vitro effects as TPI-2, designated as TPI-3 (Figure 3). Both small molecules resulted in increased activity of all three MKP1 substrates (ERK, p38 MAPK, and JNK) in Jurkat leukemia cells. In addition, both inhibitors increased cancer cell apoptosis in several cancer cell lines, including melanoma (WM9 and A539) and breast cancer (MCF-7) cells. While both TPI-2 and TPI-3 had similar efficacies, Kundu et al. (61) moved forward with in vivo experiments using TPI-3. TPI-3 was found to be a well-tolerated oral drug in mice that reduced tumor growth when taken with the established chemotherapeutics 5-fluorouracil (5FU) and leucovorin (LV) (62). This effect appeared to be synergistic, as tumor growth was inhibited by 78% with the use of TPI-3 and 5FU/LV compared to their individual effects on tumor growth (61). Since TPI-2 and TPI-3 have not been tested against other MKPs to determine specificity for MKP1, it is not yet established whether the actions of these compounds on tumor growth occur entirely through MKP1 inhibition.

### PAC1 (*DUSP2*)

Phosphatase of activated cells 1 (PAC1/*Dusp2*) inactivates both ERK and p38 MAPK (63) and is predominately expressed in hematopoietic tissues (64). PAC1 is overexpressed in inflammatory bowel disease and rheumatoid arthritis (Table 1), and mice lacking the expression of PAC1/*Dusp2* exhibit impaired inflammatory responses and protection from arthritis. Therefore, one useful application of PAC-1 inhibition will be to curtail its activity as a therapeutic for inflammatory diseases such as rheumatoid arthritis.

### Salubrinal

Salubrinal (Figure 3) is a synthetic organic compound known to inhibit the serine phosphatase protein phosphatase 1 (PP1) and eukaryotic translational initiation factor 2α (eIF2α) (65), which was used to test for inhibition against PAC1 as a potential rheumatoid arthritis treatment. Since salubrinal is also known to impact the p38 MAPK and nuclear factor κB (NF-κB) pathways (66), a genome-wide microarray was performed to analyze specific genes affected by the compound linked to DUSPs, namely PAC1 (67).

Genomic analysis using RAW 264.7 and Jurkat cells revealed that salubrinal suppressed PAC1 expression in both cell lines compared to MKP1, MKP2, and HVH3, whose expressions were only affected in one of the two cell lines. It was also found that anticollagen antibody–induced arthritis (CAIA) mice that received salubrinal showed reduced paw swelling compared to placebo-treated CAIA mice (67). The authors did not discuss salubrinal’s potential mechanism of action on PAC1 or whether salubrinal interacts directly with PAC1. Nevertheless, its ability to suppress PAC1 expression can be exploited for potential development toward arthritis treatment.

### AS077234-4

Pescini Gobert et al.’s (68) study to find MKP5 inhibitors that could reduce its effect on oligodendrocyte precursor cell differentiation revealed that their top small-molecule hit also inhibited PAC1 activity (IC_50_ = 0.48 μM). While interactions of this compound with PAC1 were not explored, this compound might have useful value as an exploratory research tool to probe the mechanism of MKP5/PAC1 action.

### MKP2 (*DUSP4*)

MKP2, encoded by *Dusp4*, is a negative regulator of all three MAPKs with a preference for ERK and JNK over p38 MAPK (69, 70). MKP2 is upregulated in a model of autoimmune encephalomyelitis, and deletion of MKP2 in mice curtails the development of the disease (Table 1). These observations suggest that MKP2 is a target for the treatment of autoimmune encephalomyelitis (71).

Park et al. (72) sought to identify small-molecule inhibitors of MKP2 using a structure-based virtual screen with docking simulation and in vitro enzymatic assays. Using the existing crystal structure of the catalytic domain of MKP2 (54), they performed a virtual screen using a docking library of 260,000 natural and synthetic compounds. Compounds were screened using Lipinski’s rule of five (73) to find those with potential physiochemical properties and nonreactive functional groups. In this screen, 150 compounds met this rule, 143 of which were commercially available for purchase for in vitro testing. The catalytic domain of MKP2 was used for enzymatic assays with these compounds using the substrate 6,8-difluoro-4-methylumbelliferyl phosphate (DiFMUP). Five compounds, labeled as Compounds 1–5, inhibited MKP2 by more than 50% at a 10-μM dose with IC_50_ values of 3.5, 6.9, 9.7, 10.2, and 10.8 μM, respectively (72). MKP7, which is highly homologous to MKP2, was also screened, and Compounds 1–3 demonstrated similar efficacies for both phosphatases, while Compounds 4–5 preferentially inhibited MKP2 (Figure 3). Structural modeling with these compounds showed that they were active-site inhibitors and that the use of pharmacophores such as benzoate, nitrobenzene, and sulfonylurea groups can serve as sufficient phosphotyrosine mimetics that mitigate the issues of being charged moieties. These compounds may serve as attractive starter compounds for further development of more efficient MKP2 inhibitors.

### MKP3 (*DUSP6*)

MKP3, encoded by *DUSP6*, is an ERK-specific phosphatase and requires ERK binding for its catalytic activation, making MKP3 one of the four inducible MKPs (45, 74). As a negative regulator of the RAS/MAPK pathway, MKP3 also mediates growth factor–receptor signaling, such as the fibroblast growth factor (FGF) receptor (75). Upregulation of MKP3 has been linked to type 2 diabetes, obesity, and multiple sclerosis (Table 1). Therefore, MKP3 has been identified as a potential therapeutic target in these diseases. It is important to note that most MKP3 inhibitors discussed below have also been identified as MKP1 inhibitors.

### NSC357756

Three MKP3 inhibitors were identified through a HTS with a computationally developed subset of the National Cancer Institute’s (NCI) compound library, the NCI Diversity Set (76). The screen used a solid-phase flow cytometry assay with a fluorescent readout for activated nuclear ERK accumulation (77). From the 1,990 compounds tested, 34 resulted in activated nuclear ERK accumulation, and 10 were subsequently confirmed as positive hits. These 10 compounds were counter-screened against other MKPs and PTPs, resulting in the identification of three hits (NSC45382, NSC295642, and NSC357756) that inhibited MKP3 by more than 50%, and of the three, NSC357756 (Figure 3) had selectivity for MKP3 over the other phosphatases and had the highest potency, with an IC_50_ of 8.0 μM (76). NSC357756 was confirmed as a MKP3 inhibitor using a technique developed by the Vogt lab known as chemical complementation (78). The chemical complementation technique allows for an unbiased assessment of MKP inhibitors without requiring any structural knowledge, binding partners, or activity to identify small-molecule hits. The authors utilized the NCI’s Developmental Therapeutics Program website to demonstrate that NSC357756 has been reported to have anticancer activity when tested against P388 leukemia, L1210 leukemia, and M5076 sarcoma cell lines. While they did not identify the mechanism of inhibition or any structural information on MKP3 binding, Vogt et al. (78) are credited with reporting the first small-molecule inhibitor of an MKP, and specifically of MKP3, with low micromolar potency.

### NSC95397

Using compounds known to inhibit Cdc25, Vogt et al. (79) went on to screen for their potential to inhibit either MKP3 or MKP1. This screen was once again performed by employing their chemical complementation technique. From the four inhibitors used, NSC95397 was identified to inhibit both MKP3 and MKP1 with an IC_50_ of 13 μM (Figure 3). In vitro data showed that NSC95397 preferentially inhibited MKP3 (IC_50_ = 25 ± 12 μM) over MKP1 (IC_50_ = 65 ± 21 μM). This relatively weak level of MKP1 and MKP3 inhibition likely would have been missed using in vitro enzymatic assays, which often use truncated enzymes and artificial small-molecule substrates versus physiological endogenous substrates (79), demonstrating the value of this chemical complementation screen over traditional assays. NSC95397 has been used in vivo to treat mice for IL-10-dependent colitis by inhibiting MKP1 and MKP3 (80). Dexamethasone protects cancer cells from undergoing apoptosis following treatment with the chemotherapeutic agent paclitaxel by inducing MKP1 expression. The protection from paclitaxel-induced apoptosis by dexamethasone is abrogated by treatment of cancer cells with NSC95397 and similarly by small interfering RNA knockdown of MKP1 (79, 81). These observations suggest some level of on-target MKP1 engagement by NSC95397 in cells. This implies that in cancers that express high levels of MKP1, sensitization to the apoptosis-inducing effects of antineoplastic agents could be conferred through combination therapy with MKP1 inhibitors.

### BCI

A zebrafish chemical screen that sought to identify small-molecule inhibitors that modulated FGF signaling led to the discovery of a MKP3 inhibitor (82). The screen was conducted using a transgenic zebrafish line with green fluorescent protein under the control of the fibroblast growth factor. From a pool of 5,000 compounds, BCI [(*E*)-2-benzylidene-3-(cyclohexylamino)-2,3-dihydro-1*H*-inden-1-one] (Figure 3) was identified as the top hit, and probing the MAPK pathway identified the target of BCI as MKP3. Molina et al. (82) showed that BCI blocked MKP3 activity in both cultured cells and embryos, where MKP3 inhibition enhanced FGF signaling in the heart. Cell-based studies indicated that while BCI was an effective MKP3 inhibitor, it also inhibited MKP1 to equivalent levels, with IC_50_ values for MKP3 and MKP1 of 11.5 ± 2.8 μM and 12.3 ± 3.4 μM, respectively. Molina et al. performed modeling to predict how BCI interacted with MKP3, which showed that BCI binds at an allosteric site within the MKP3 PTP domain and binds to the low-activity conformation of MKP3 in the WPD loop with residues within the α7 helix away from the active-site cysteine (82). Thus, BCI was designated as an allosteric inhibitor of MKP3. This study was a springboard for the development of additional and potentially more potent BCI analogs (83). Although BCI was still the most potent inhibitor, the use of other analogs revealed that these molecules interacted with residues distant from the active-site cysteine, supporting the notion that BCI and the analogs were allosteric inhibitors of MKP3 and, likely, MKP1. BCI has been used in studies to inhibit MKP3 activity in instances where MKP3 overexpression confers drug resistance to cancer treatments and/or increased growth and proliferation of cancer cells (84, 85). However, it is important to point out that it remains to be formally demonstrated using structural biology approaches as to whether the site of BCI and its analogs truly interact at sites distant from the catalytic cysteine residue.

### Adociaquinone B

An in vitro enzymatic assay designed to discover Cdc25 inhibitors used purified PTP domains of both MKP3 and MKP1 as counter-screens. This strategy led to the identification of a quinone derivative, adociaquinone B (Figure 3), as an inhibitor of MKPs (86). There were two top hits for the MKPs: adociaquinone B, which inhibited MKP3 and MKP1 with IC_50_ values of 1.53 μM and 1.10 μM, respectively, and the derivative Compound 20 that was slightly more potent against MKP1 (IC_50_ = 0.82 μM) over MKP3 (IC_50_ = 1.35 μM). Despite the impressive potency for the MKPs, the IC_50_ values of these compounds toward Cdc25B were still four- to tenfold higher, indicating that they are more Cdc25B-specific inhibitors. No insights were provided on whether adociaquinone B was an active site inhibitor.

### MKP5 (*DUSP10*)

MKP5 has been shown to have preferential catalytic activity against p38 MAPK and JNK compared to ERK (87, 88), and it is ubiquitously expressed at low levels in many tissues. However, it is more abundant in skeletal muscle, liver, and hemopoietic systems (89). MKP5’s overall importance and function has been explored through the use of MKP5-deficient mice, revealing phenotypes such as enhanced innate immunity, reduced lipopolysaccharide (LPS)-induced vascular injury, increased regenerative myogenesis in mice with muscular dystrophy, and impaired pulmonary fibrosis (Table 1). These observations have supported the notion that MKP5 inhibition may provide novel therapeutic avenues for the treatment of inflammatory diseases, dystrophic muscle disease, and idiopathic pulmonary fibrosis.

### AS077234-4

Using multiple reagents to stimulate oligodendrocyte precursor (OPC) differentiation, Pescini Gobert et al. (68) identified MKP5 as one of three repressor genes that impacted OPC differentiation. To further elucidate MKP5’s role in OPC differentiation, the authors used an inhibitor of MKP5 called AS077234–4 that inhibited MKP5 with an IC_50_ of 0.7 μM. This inhibitor was found to be selective for MKP5 against most of the PTPs tested, except PAC1, for which it proved to be more potent (IC_50_ = 0.48 μM). Treatment of rat OPC cells with AS077234–4 showed a concentration-dependent increase in the differentiation marker proteins as is seen with MKP5 knockdown. Unfortunately, the chemical structure of AS077234–4 was not reported, and little information was provided about the inhibitor’s mechanism of action or whether it inhibited other MKPs or lead to an upregulation of the MAPKs, as would be anticipated for a MKP5-specific inhibitor.

### Compound 1

A HTS using the PTP domain of MKP5 identified a small-molecule allosteric inhibitor of MKP5 (Figure 3) with low micromolar potency (90). To identify inhibitors that interacted with MKP5 outside of the active site, an 11-amino-acid doubly phosphorylated p38 MAPK peptide mimetic of the activation loop was used, as opposed to the typically used small-molecule substrates such as *para*-nitrophenyl phosphate (pNPP) and DiFMUP. From the 162,000 compounds tested in the screen, when the top 27 hits were tested against striatal-enriched protein tyrosine phosphatase (STEP-46) and PTP1B as counter-screens, a top hit emerged for MKP5 (IC_50_ = 3.9 μM) with an IC_50_ more than a hundredfold higher than those for the other two phosphatases. Furthermore, when tested against MKP1, MKP7, and MKP3, the small-molecule inhibitor designated as Compound 1 was selective for MKP5, with the other MKPs having IC_50_ values more than 16-fold greater than that of MKP5. Compound 1 was also tested with full-length MKP5 in a similar in vitro assay and had roughly the same effect as with the catalytic domain alone. Gannam et al. (90) solved the cocrystal structure of MKP5 bound with Compound 1 (6MC1), which allowed for further insights into how Compound 1 inhibited MKP5. This cocrystal structure represented the first-ever MKP bound to a small molecule. The cocrystal structure revealed that Compound 1 bound to MKP5 about 8 Å away from the catalytic cysteine and that key interacting residues were not a part of the active site, indicating that Compound 1 is an allosteric inhibitor. In addition, mutational analysis revealed that residues that were required for Compound 1 binding inactivated MKP5 activity. Interestingly, these key Compound 1–binding residues were conserved among all the catalytically active MKP family members. Comparing the apo-crystal structure of MKP5 (1ZZW) (52) to the cocrystal structure bound to Compound 1 demonstrated that there was an 18% decrease in the catalytic pocket volume. This decreased pocket volume impacts substrate access to the active site, which negatively impacts MKP5 catalysis (90). Additionally, the cocrystal structure was used to model MAPK binding, which predicted that there is a misorientation of the phosphorylated activation loop of the incoming MAPK as a result of active site pocket distortion. Finally, modeling experiments also implied the possibility that Compound 1, when bound to this allosteric site, could interfere with binding of the MAPK substrate to MKP5. Collectively, this three-mode mechanism of inhibition provides a compelling mechanistic basis for how the allosteric binding of Compound 1 inhibits MKP5 catalysis.

To support these in vitro data, Compound 1 was used in cell-based assays. Administration of Compound 1 into mouse fibroblasts led to increased p38 MAPK and JNK, but not ERK, phosphorylation (90). Similar results on MAPK activity were seen when myoblasts were treated with Compound 1 in addition to an enhanced muscle differentiation phenotype, which phenocopied the effects previously reported for myoblasts derived from MKP5-deficient mice (37). These results demonstrated that Compound 1 can recapitulate the MKP5-deficient genetic phenotype, supporting the interpretation that it exhibits MKP5 specificity. Additionally, to substantiate a previously reported link between MKP5 and the proinflammatory cytokine TGF-β’s role in lung fibrosis (91), MKP5-deficient and wild-type fibroblasts were treated with Compound 1. MKP5-deficient mice were shown to exhibit reduced phosphorylation of the TGF-β receptor transcriptional target Smad2 (91). Similarly, treatment of wild-type fibroblasts with Compound 1 resulted in decreased Smad2 phosphorylation and a concomitant increase in p38 MAPK and JNK phosphorylation, and there was no additional effect in MKP5-deficient fibroblasts (90). These results collectively demonstrated that Compound 1 inhibits MKP5 and thus represents a potential therapeutic for fibrotic tissue disease by blocking excessive TGF-β signaling. While there is room for further analysis of Compound 1’s effect on the TGF-β/MKP5 pathway, it is emerging as an important tool compound that can be used to further investigate the role of MKP5 in various physiological and pathophysiological settings.

To date, Compound 1 represents the only experimentally established allosteric inhibitor of an MKP. While BCI has been described as an allosteric inhibitor, this has only been inferred through computational modeling (82). In contrast, Compound 1’s allosteric mode of inhibition is supported by its cocrystal structure with MKP5, and this is corroborated by enzymatic kinetic analyses (90). Further development of Compound 1 is needed to improve on both its potency and solubility. Nevertheless, it will serve as an excellent exploratory tool to study MKP5-mediated signaling. Given MKP5’s involvement in promoting fibrosis in tissues such as skeletal muscle (37), lung (91) and the heart (35), the future development of therapeutic strategies targeting MKP5 for the treatment of fibrosis could be very impactful.

### MKP7 (*DUSP16*)

MKP7 inactivates both p38 MAPK and JNK and is capable of binding to all three MAPKs, including ERK (92, 93). While MKP7 is one of the less-explored MKPs for its role in disease, it has been shown to confer resistance to antineoplastic-mediated cell death in certain cancers (94, 95). Also, due to its negative effect on JNK activity, it has been implicated in the regulation of T helper cell differentiation (96) and therefore potentially has a role in inflammatory diseases (97). Thus, there is much to be gained from small-molecule inhibition of MKP7, both as an exploratory tool to further understand its mechanism of action and for potential cancer therapeutics.

Park et al. (98) employed a structure-based virtual compound screen for MKP7 inhibitors. At the time of the study, the crystal structure of MKP7 had yet to be solved, and so the computational three-dimensional structure used the highly homologous crystal structure of MKP4 as a template (46). The same 260,000-compound library from the MKP2 screen (72) was used, and 148 commercially available compounds were tested in an in vitro assay against the purified PTP domain of MKP7. Seven top hits, labeled Compounds 1–7, were identified that inhibited MKP7 by more than 50% at a concentration of 20 μM, with IC_50_ values ranging from 1.3 to 21.3 μM (98). When tested against other phosphatases, MKP5 and DUSP25, which both have significant sequence similarity to MKP7, Compounds 1, 2, 4, and 7 (Figure 3) were about twofold less potent against the other two MKPs. However, Compound 3 had greater inhibition against both MKP5 (IC_50_ = 1.7 ± 0.5 μM) and DUSP25 (IC_50_ = 0.2 ± 0.1 μM) than MKP7 (IC_50_ = 4.8 ± 1.2 μM) (98). Docking simulations indicated that Compounds 1–7 had electrostatic interactions with key residues in MKP7’s active site, including Cys244 and Arg250, indicating that these were active-site inhibitors.

## PROSPECTS OF MKP INHIBITOR DEVELOPMENT

From the MKP small-molecule inhibitors described herein, it is apparent that there has been advancement in the development of MKP inhibitors. However, there remain significant hurdles that need to be overcome to move these inhibitors from low-micromolar to nanomolar potency with high specificity. These advancements will require efforts that employ a shift in the strategies used for MKP inhibitor discovery. Typically, the catalytic domains of the MKPs have been used in HTSs, and this has proven to be quite efficient. However, in cases where sequence and structure similarities are highly conserved, as is the case with the MKPs, full-length proteins will be more advantageous. Using full-length proteins in these enzymatic assays may capitalize on the structural differences not evident within the PTP domain of these MKPs. This would potentially address the issue of MKP inhibitor selectivity and could open the door to the discovery of more allosteric inhibitors. This approach of using full-length enzymes has proven particularly advantageous in the discovery of the SHP2 allosteric inhibitor, SHP099 (99). However, the challenge in using full-length proteins is the need to express and purify large amounts of protein for enzymatic assays. With the advent of expression technologies in multiple organism systems, fusion-protein expression systems, and expression with chaperone proteins, isolation of full-length MKPs for HTS is more than likely attenable (100, 101).

Small molecules such as pNPP and DiFMUP have been utilized as substrates in enzymatic assays for decades due to their cost effectiveness, ease of use, and efficient modes of output such as fluorescence readouts (102, 103). However, these small molecules only interact with the MKPs at the active-site cysteine and have very little interaction with other residues. Given that the MKPs share highly similar active sites, there is likely to be substantial lack of selectivity in compound screens that use these small molecules as substrates. As shown with the discovery of the MKP5 allosteric inhibitor, Compound 1, the use of a phosphorylated p38 MAPK peptide mimetic allows for greater interaction between MKP5 and the substrate that extends outside of the active site (90). These extended surface interactions mitigate nonspecific inhibitor hits and facilitate the identification of allosteric inhibitors. Shifting toward phospho-peptide substrate mimetics in HTS compound assays has the likelihood to usher in the next generation of highly specific MKP inhibitors.

Another strategy could employ the use of bidentate inhibitors capable of binding to an enzyme at both the active site and a nonconserved binding region of the enzyme, leading to more effective and specific inhibition (104). These types of inhibitors were applied to the inhibition of PTP1B (105) and have led to the discovery of several PTP1B bidentate inhibitors (106). There have also been successful bidentate inhibitors developed against other PTPs such as SHP2 with the 11a-1 compound (107). The concept of bidentate inhibitors is quite attractive for MKP inhibition due to their conserved catalytic site. Since MKPs also interact with their substrates outside of their active site at MAPK docking motifs, these regions might also be leveraged as second site locations for bidentate inhibitor interactions (108). This two-pronged approach of both active site and alternative site inhibitor binding can lead to substantially higher potency inhibition of the MKPs while also conferring higher specificity.

## ALTERNATIVE APPROACHES TO MKP INHIBITION

While small-molecule inhibition has been the most common approach for disease therapeutic development, there are other modes of inhibition that may prove useful in order to overcome the challenges faced by small-molecule inhibitors to the MKPs.

Antibody-based therapy to regulate protein function is an emerging strategy that has had some success with the development of monoclonal antibodies against the phosphatases of regenerating liver (PRLs). Generation of PRL-specific mouse and chimeric antibodies led to reduced metastatic tumor formation derived from several cancer cell lines overexpressing PRL-3 (109). Antibody function was deemed specific, since there was no effect in tumors derived from cancer cells not expressing PRL-3. Also, while permeating the cell membrane is challenging for an antibody, the use of bioadaptors has helped to address these issues, and using native bioadaptor antibodies has proven successful in the inhibition of PTP1B (110).

Antisense gene therapy continues to be of interest as a mode of protein inhibition. An antisense oligonucleotide (ASO) to PTP1B, when given to obese or type 2 diabetic mice, caused a reduction in PTP1B expression in both the liver and adipose tissue, which correlated with improved insulin signaling and sensitivity (111). This PTP1B ASO led to a second-generation ASO known as IONIS-PTP-1B_Rx_ that is currently in Phase II clinical trials for the treatment of type 2 diabetes in overweight patients (112). Given that a number of MKPs are overexpressed in cancer, immunological diseases, and certain tissues that promote fibrosis (Table 1), strategies to inactivate the MKPs using ubiquitin-mediated proteasomal approaches offer a potentially attractive path. The success of using proteolysis-targeting chimeras (PROTACs) against SHP2 for the treatment of cancer has been demonstrated (113). Thus, therapeutic techniques utilizing PROTACS that are designed to be specific to the MKPs may also be an appealing approach.

## FUTURE PERSPECTIVES

While there is still much unexplored territory in the area of MKP inhibition, there have been great strides in small-molecule inhibitor development that have demonstrated that specific and effective inhibition of MKPs is possible. Small-molecule MKP inhibitors have been useful in their application as tools toward a better understanding of MKP signaling and function. The next steps will likely continue to be challenging, as the development of highly potent and orally bioavailable MKP inhibitors is needed to provide preclinical proof-of-principle for the use of MKP inhibitors in the treatment of disease. This next milestone will be an important accomplishment that will provide a path forward toward new therapeutics for the treatment of disease.

## Figures and Tables

**Table 1:** MKPs and disease

Gene name	Name(s)^a^	MAPK substrate(s)	Disease implications (references)^b^
***DUSP1***	MKP1/CL100	p38 MAPK, JNK, ERK	Breast (27, 114), lung (29), and prostate (115) cancer Obesity/type 2 diabetes (116, 117)
***DUSP2***	PAC1	p38 MAPK, ERK	Arthritis (118) Ovarian cancer (119)
***DUSP4***	MKP2	p38 MAPK, JNK, ERK	Autoimmune encephalomyelitis (120) Sepsis (121) Melanoma (122)
***DUSP5***	HVH3	ERK	Unknown
***DUSP6***	MKP3/PYST1	ERK	Obesity/type 2 diabetes (123, 124) Multiple sclerosis (125) Cardiac myopathy (126)
***DUSP7***	MKPX/PYST2	ERK	Acute and myeloid leukemia (127)
***DUSP8***	HVH5	p38 MAPK, JNK	Cardiomyopathy (128)
***DUSP9***	MKP4/PYST3	p38 MAPK, ERK	Insulin resistance/type 2 diabetes (129)
***DUSP10***	MKP5	p38 MAPK, JNK	Pulmonary fibrosis (91) Duchenne muscular dystrophy (37) Acute myeloid leukemia (130) Colorectal cancer (131)
***DUSP16***	MKP7	p38 MAPK, JNK	Colorectal, gastric, and breast cancer (94) Hepatocellular carcinoma (95)

a Only active MKPs are listed.

b This is not an exhaustive list of all diseases in which MKPs have a role but rather a few poignant examples in which MKP activity/expression is positively correlated to disease progression.

Abbreviations: ERK, extracellular signal–regulated kinase; JNK, c-Jun N-terminal kinase; MAPK, mitogen-activated protein kinase; MKP, MAPK phosphatase.
